# Elevated CSF and plasma complement proteins in genetic frontotemporal dementia: results from the GENFI study

**DOI:** 10.1186/s12974-022-02573-0

**Published:** 2022-09-05

**Authors:** Emma L. van der Ende, Carolin Heller, Aitana Sogorb-Esteve, Imogen J. Swift, David McFall, Georgia Peakman, Arabella Bouzigues, Jackie M. Poos, Lize C. Jiskoot, Jessica L. Panman, Janne M. Papma, Lieke H. Meeter, Elise G. P. Dopper, Martina Bocchetta, Emily Todd, David Cash, Caroline Graff, Matthis Synofzik, Fermin Moreno, Elizabeth Finger, Raquel Sanchez-Valle, Rik Vandenberghe, Robert Laforce, Mario Masellis, Maria Carmela Tartaglia, James B. Rowe, Chris Butler, Simon Ducharme, Alexander Gerhard, Adrian Danek, Johannes Levin, Yolande A. L. Pijnenburg, Markus Otto, Barbara Borroni, Fabrizio Tagliavini, Alexandre de Mendonça, Isabel Santana, Daniela Galimberti, Sandro Sorbi, Henrik Zetterberg, Eric Huang, John C. van Swieten, Jonathan D. Rohrer, Harro Seelaar, the Genetic Frontotemporal Dementia Initiative (GENFI), Sónia Afonso, Maria Rosario Almeida, Sarah Anderl-Straub, Christin Andersson, Anna Antonell, Silvana Archetti, Andrea Arighi, Mircea Balasa, Myriam Barandiaran, Nuria Bargalló, Robart Bartha, Benjamin Bender, Alberto Benussi, Luisa Benussi, Valentina Bessi, Giuliano Binetti, Sandra Black, Martina Bocchetta, Sergi Borrego-Ecija, Jose Bras, Rose Bruffaerts, Marta Cañada, Valentina Cantoni, Paola Caroppo, David Cash, Miguel Castelo-Branco, Rhian Convery, Thomas Cope, Giuseppe Di Fede, Alina Díez, Diana Duro, Chiara Fenoglio, Camilla Ferrari, Catarina B. Ferreira, Nick Fox, Morris Freedman, Giorgio Fumagalli, Alazne Gabilondo, Roberto Gasparotti, Serge Gauthier, Stefano Gazzina, Giorgio Giaccone, Ana Gorostidi, Caroline Greaves, Rita Guerreiro, Tobias Hoegen, Begoña Indakoetxea, Vesna Jelic, Hans-Otto Karnath, Ron Keren, Tobias Langheinrich, Maria João Leitão, Albert Lladó, Gemma Lombardi, Sandra Loosli, Carolina Maruta, Simon Mead, Gabriel Miltenberger, Rick van Minkelen, Sara Mitchell, Katrina Moore, Benedetta Nacmias, Jennifer Nicholas, Linn Öijerstedt, Jaume Olives, Sebastien Ourselin, Alessandro Padovani, Georgia Peakman, Michela Pievani, Cristina Polito, Enrico Premi, Sara Prioni, Catharina Prix, Rosa Rademakers, Veronica Redaelli, Tim Rittman, Ekaterina Rogaeva, Pedro Rosa-Neto, Giacomina Rossi, Martin Rosser, Beatriz Santiago, Elio Scarpini, Sonja Schönecker, Elisa Semler, Rachelle Shafei, Christen Shoesmith, Miguel Tábuas-Pereira, Mikel Tainta, Ricardo Taipa, David Tang-Wai, David L. Thomas, Paul Thompson, Hakan Thonberg, Carolyn Timberlake, Pietro Tiraboschi, Emily Todd, Philip Van Damme, Mathieu Vandenbulcke, Michele Veldsman, Ana Verdelho, Jorge Villanua, Jason Warren, Carlo Wilke, Ione Woollacott, Elisabeth Wlasich, Miren Zulaica

**Affiliations:** 1 Alzheimer Center Rotterdam and Department of Neurology, Erasmus University Medical Center, PO Box 2040, 3000 CA Rotterdam, The Netherlands; 2 UK Dementia Research Institute at University College London, UCL Queen Square Institute of Neurology, London, UK; 3 Dementia Research Centre, Department of Neurodegenerative Disease, UCL Queen Square Institute of Neurology, University College London, London, UK; 4 Department of Pathology, University of California San Francisco, San Francisco, USA; 5 Center for Alzheimer Research, Division of Neurogeriatrics, Department of Neurobiology, Care Sciences and Society, Bioclinicum, Karolinska Institutet, Solna, Sweden; 6 Unit for Hereditary Dementias, Theme Aging, Karolinska University Hospital, Solna, Sweden; 7 German Center for Neurodegenerative Diseases (DZNE), Tübingen, Germany; 8 Department of Neurodegenerative Diseases, Hertie Institute for Clinical Brain Research and Center of Neurology, University of Tübingen, Tübingen, Germany; 9 Cognitive Disorders Unit, Department of Neurology, Hospital Universitario Donostia, San Sebastian, Gipuzkoa Spain; 10 Neuroscience Area, Biodonostia Health Research Institute, San Sebastian, Gipuzkoa Spain; 11 Department of Clinical Neurological Sciences, University of Western Ontario, London, ON Canada; 12 Alzheimer’s Disease and Other Cognitive Disorders Unit, Neurology Service, Hospital Clinic, IDIBAPS, University of Barcelona, Barcelona, Spain; 13 Laboratory for Cognitive Neurology, Department of Neurosciences, Leuven Brain Institute, KU Leuven, Louvain, Belgium; 14 Clinique Interdisciplinaire de Mémoire, Département Des Sciences Neurologiques, CHU de Québec, Université Laval, Québec, Canada; 15 Sunnybrook Research Institute, Toronto, ON Canada; 16 Tanz Centre for Research in Neurodegenerative Disease, University of Toronto, Toronto, ON Canada; 17 Cambridge University Centre for Frontotemporal Dementia, University of Cambridge, Cambridge, UK; 18 Nuffield Department of Clinical Neurosciences, Medical Sciences Division, University of Oxford, Oxford, UK; 19 McConnell Brain Imaging Centre, Montreal Neurological Institute and McGill University Health Centre, McGill University, Montreal, Québec Canada; 20 Department of Nuclear Medicine and Geriatric Medicine, University Hospital Essen, Essen, Germany; 21 Division of Neuroscience and Experimental Psychology, Wolfson Molecular Imaging Centre, University of Manchester, Manchester, UK; 22 Neurologische Klinik Und Poliklinik, Ludwig-Maximilians-Universität München, Munich, Germany; 23 German Center for Neurodegenerative Diseases, Munich, Germany; 24 Munich Cluster for Systems Neurology (SyNergy), Munich, Germany; 25 Alzheimer Center Amsterdam, Department of Neurology, Amsterdam Neuroscience, Vrije Universiteit Amsterdam, Amsterdam UMC, Amsterdam, The Netherlands; 26 Department of Neurology, Universität Ulm, Ulm, Germany; 27 Centre for Neurodegenerative Disorders, Department of Clinical and Experimental Sciences, University of Brescia, Brescia, Italy; 28 Fondazione IRCCS Istituto Neurologico Carlo Besta, Milan, Italy; 29 Faculdade de Medicina da Universidade de Lisboa, Lisbon, Portugal; 30 Center for Neuroscience and Cell Biology, Faculty of Medicine, University of Coimbra, Coimbra, Portugal; 31 Fondazione IRCCS, Ospedale Maggiore Policlinico, Neurodegenerative Diseases Unit, Milan, Italy; 32 University of Milan, Centro Dino Ferrari, Milan, Italy; 33 Department of Neurofarba, University of Florence, Florence, Italy; 34 Department of Psychiatry and Neurochemistry, Sahlgrenska Academy at the University of Gothenburg, Mölndal, Sweden

**Keywords:** Biomarker, Complement, Frontotemporal dementia, Neuroinflammation

## Abstract

**Background:**

Neuroinflammation is emerging as an important pathological process in frontotemporal dementia (FTD), but biomarkers are lacking. We aimed to determine the value of complement proteins, which are key components of innate immunity, as biomarkers in cerebrospinal fluid (CSF) and plasma of presymptomatic and symptomatic genetic FTD mutation carriers.

**Methods:**

We measured the complement proteins C1q and C3b in CSF by ELISAs in 224 presymptomatic and symptomatic *GRN, C9orf72* or *MAPT* mutation carriers and non-carriers participating in the Genetic Frontotemporal Dementia Initiative (GENFI), a multicentre cohort study. Next, we used multiplex immunoassays to measure a panel of 14 complement proteins in plasma of 431 GENFI participants. We correlated complement protein levels with corresponding clinical and neuroimaging data, neurofilament light chain (NfL) and glial fibrillary acidic protein (GFAP).

**Results:**

CSF C1q and C3b, as well as plasma C2 and C3, were elevated in symptomatic mutation carriers compared to presymptomatic carriers and non-carriers. In genetic subgroup analyses, these differences remained statistically significant for *C9orf72* mutation carriers. In presymptomatic carriers, several complement proteins correlated negatively with grey matter volume of FTD-related regions and positively with NfL and GFAP. In symptomatic carriers, correlations were additionally observed with disease duration and with Mini Mental State Examination and Clinical Dementia Rating scale® plus NACC Frontotemporal lobar degeneration sum of boxes scores.

**Conclusions:**

Elevated levels of CSF C1q and C3b, as well as plasma C2 and C3, demonstrate the presence of complement activation in the symptomatic stage of genetic FTD. Intriguingly, correlations with several disease measures in presymptomatic carriers suggest that complement protein levels might increase before symptom onset. Although the overlap between groups precludes their use as diagnostic markers, further research is needed to determine their potential to monitor dysregulation of the complement system in FTD.

**Supplementary Information:**

The online version contains supplementary material available at 10.1186/s12974-022-02573-0.

## Background

Frontotemporal dementia (FTD) is a common form of young-onset dementia and is frequently caused by autosomal dominant genetic mutations in progranulin (*GRN)*, chromosome 9 open reading frame 72 (*C9orf72*) or microtubule-associated protein tau (*MAPT*) [1, 2]. Accumulating evidence suggests a role for neuroinflammation in FTD, although the timing and exact contribution to disease pathogenesis remains unclear [3]. Fluid biomarkers that reflect neuroinflammation in vivo could be valuable for clinical practice and therapeutic trials. Previous studies aiming to identify such biomarkers, including cytokines and microglial markers, have yielded somewhat inconsistent results [4–13] 

The complement system is a key component of innate immunity and comprises a cascade of protein reactions which ultimately result in opsonisation and lysis of potential pathogens, recruitment of immune cells to create a pro-inflammatory environment, and clearance of apoptotic cells [14]. Complement proteins are also involved in microglia-mediated synaptic pruning in both the developing and adult brain [15, 16], and aberrant activation of the complement cascade is thought to play a central role in synaptic degeneration across neurodegenerative diseases [17–20]. In line with this, *GRN-/-* mice display excessive complement activation and synaptic pruning, whereas deletion of the complement genes *C1q* and *C3b* mitigates synapse loss and neurodegeneration [21, 22]. Complement proteins in cerebrospinal fluid (CSF) and blood are differentially regulated in Alzheimer’s disease (AD) [23–28] and other neurodegenerative diseases compared to controls [29–32], but they have not been thoroughly investigated in FTD. Promisingly, an inverse correlation was found between CSF C1q and C3b levels and Mini Mental State Examination (MMSE) score in a small series of *GRN* mutation carriers [21].

In the present study, we measured a range of complement proteins in CSF and plasma of presymptomatic and symptomatic genetic FTD mutation carriers participating in the international Genetic FTD Initiative (GENFI). To determine their value as disease progression biomarkers, we correlated complement levels with corresponding clinical and neuroimaging measures. Finally, we explored their relationship with biomarkers that reflect neuro-axonal degeneration (neurofilament light chain, NfL) [33, 34] and astrogliosis (glial fibrillary acidic protein, GFAP) [35, 36].

## Methods

### Subjects

Subjects were recruited from 19 centres collaborating in GENFI, a longitudinal cohort study which follows patients with genetic FTD due to a mutation in *GRN*, *C9orf72* or *MAPT* and their 50% at-risk family members (either presymptomatic mutation carriers or non-carriers) [37]. Participants underwent an annual assessment as previously described [37], which includes a brief medical history, neurological and neuropsychological examination, magnetic resonance imaging (MRI) of the brain, and collection of blood and CSF. Clinical researchers were blinded to the genetic status of at-risk individuals unless they had undergone predictive testing. Subjects with known auto-immune diseases were excluded from the current study as complement levels could be affected [38].

CSF samples were available in 104 presymptomatic (46 *GRN,* 42 *C9orf72,* 16 *MAPT*) and 46 symptomatic mutation carriers (11 *GRN*, 28 *C9orf72,* 7 *MAPT*) and 74 healthy non-carriers. Plasma samples were available in 215 presymptomatic (88 *GRN*, 80 *C9orf72*, 47 *MAPT*) and 104 symptomatic mutation carriers (36 *GRN,* 47 *C9orf72*, 21 *MAPT*) and 112 non-carriers (Table 1). 174 subjects were included in both the CSF and plasma cohorts.

**Table 1:** Subject characteristics for (a) CSF and (b) plasma measurements

(a) CSF cohort								
	Non-carriers	Presymptomatic carriers			Symptomatic carriers^a^			*p*
N	74	104	46					
Sex, male (%)	34 (46%)	43 (41%)	28 (61%)	0.085				
Age at collection, years Years	47 (39–58)	46 (35–56)	63 (55–69)	< 0.001				
MMSE (*n* = 219)	30 (29–30)	30 (29–30)	26 (24–29)	< 0.001				
CDR® + NACC FTLD-SB (*n* = 185)	0 (0–0)	0 (0–0)	9 (2–13)	< 0.001				
*Per genotype*		*GRN*	*C9orf72*	*MAPT*	*GRN*	*C9orf72*	*MAPT*	
N		46	42	16	11	28	7	-
Age at collection, years		54 (42–59)	43 (33–53)	42 (34–46)	67 (61–70)	60 (55–72)	59 (52–64)	< 0.001^b^
Age at symptom onset, years	–	–	–	–	64 (54–67)	56 (49–62)	55 (52–56)	0.141
Disease duration, years	–	–	–	–	2.5 (1.0–4.3)	4.1 (2.1–8.0)	2.6 (0.4–8.0)	0.229

(b) Plasma cohort								
	Non-carriers	Presymptomatic carriers			Symptomatic carriers^c^			*p*
N	112	215			104			
Sex, male (%)	49 (44%)	79 (37%)			64 (62%)			< 0.001
Age at collection, years Years	50 (39–60)	45 (35–55)			63 (58–69)			< 0.001
MMSE (*n* = 405)	30 (29–30)	30 (29–30)			25 (20–28)			< 0.001
CDR® + NACC FTLD-SB (*n* = 329)	0 (0–0)	0 (0–0)			8 (3–14)			< 0.001
*Per genotype*		*GRN*	*C9orf72*	*MAPT*	*GRN*	*C9orf72*	*MAPT*	
N		88	80	47	36	47	21	
Age at collection, years		51 (39–59)	44 (34–53)	40 (33–46)	64 (59–68)	66 (59–72)	58 (52–63)	< 0.001^b^
Age at symptom onset, years	–	–	–	–	60 (55–66)	59 (55–66)	53 (47–57)	< 0.001^d^
Disease duration, years	–	–	–	–	2.6 (1.8–4.2)	5 (2.6–6.6)	5.6 (1.5–6.8)	0.002^e^

Continuous variables are expressed as median (interquartile range) and were compared between groups using Kruskal–Wallis tests. Sex distributions were compared between groups using Chi-square tests. *MMSE*  Mini Mental State Examination, *CDR*  Clinical Dementia Rating scale, *SB*  sum of boxes

^a^Phenotypes: behavioural variant FTD (bvFTD) (*n* = 32), primary progressive aphasia (PPA) (*n* = 5), FTD with amyotrophic lateral sclerosis (ALS) (*n* = 3), ALS without FTD (*n* = 3), progressive supranuclear palsy (PSP) (*n* = 1), memory-predominant FTD (*n* = 1), dementia not otherwise specified (*n* = 1)

^b^Symptomatic mutation carriers were older than presymptomatic carriers in all genetic subgroups. ^c^Phenotypes: bvFTD (*n* = 78), PPA (*n* = 16), FTD-ALS (*n* = 2), ALS without FTD (*n* = 5), PSP (*n* = 1), memory-predominant FTD (*n* = 1), dementia not otherwise specified (*n* = 1)

^d^Symptomatic *MAPT* mutation carriers were younger at symptom onset than *C9orf72* (*p* = 0.004) and *GRN* mutation carriers (*p* = 0.002)

^e^Symptomatic *C9orf72* mutation carriers had a longer disease duration than symptomatic *GRN* carriers at sample collection

Mutation carriers were considered symptomatic if they fulfilled international consensus criteria for behavioural variant FTD [39], primary progressive aphasia [40] or amyotrophic lateral sclerosis (ALS) [41]. Disease duration was defined based on when the primary caregiver first noted symptoms. Global cognition was scored using the MMSE and Clinical Dementia Rating scale® plus NACC FTLD sum of boxes (CDR® + NACC FTLD-SB) [42], collected within 6 months of CSF or plasma sampling.

T1-weighted MRI on 3 Tesla scanners was obtained within 6 months of sample collection using a standardised GENFI protocol. T1-weighted volumetric MRI scans were parcellated into brain regions as previously described [37], using an atlas propagation and fusion strategy to generate volumes of the whole brain (WBV), frontal, temporal, parietal and occipital lobes, insula and cingulate gyrus. Brain volumes were expressed as a percentage of total intracranial volume (TIV), computed with SPM12 running under Matlab R2014b (Math Works, Natick, MA, USA) [43].

### Sample collection and laboratory methods

CSF was collected by lumbar puncture in polypropylene tubes, and blood was collected by venepuncture in EDTA tubes. Samples were centrifuged and stored at -80 °C until use according to a standardised GENFI protocol.

All CSF and plasma measurements were performed in duplicate. The mean duplicate coefficient of variation (CV) was below 10% for all analytes; samples with a CV > 20% were re-measured or excluded. For sample concentrations outside of the range of quantification, we imputed the lower or upper limits of quantification (LLOQ and ULOQ) (Additional file 1: Table S1).

CSF complement proteins C1q and C3b were measured using the ELISA kits Human Complement C1q (ab170246) and Human Complement C3b (ab195461) from Abcam (Boston, MA, USA) according to the manufacturer’s instructions. Plates were read on a SpectraMax M2 plate reader (Molecular Devices, San Jose, CA). CSF NfL was measured using the Simoa NF-Light Advantage Kit from Quanterix (Billerica, MA, USA) on a Simoa HD-1 analyzer instrument according to the manufacturer’s instructions.

Plasma complement proteins were measured using the multiplex Human Complement Magnetic Bead Panel 1 (complement factors C2, C4b, C5, C5a, C9, factor D, mannose-binding lectin, and factor I) and Human Complement Magnetic Bead Panel 2 (C1q, C3, C3b, C4, factor B, factor H) (HCMP1MAG-19 K and HCMP2MAG-19 K, respectively) kits from EMD Millipore Corporation (Billerica, MA, USA) according to the manufacturer’s instructions. Plates were analysed on a Luminex MAGPIX Instrument System (Luminex Corp, Austin, TX, USA). Plasma NfL and GFAP were measured using the multiplex Neurology 4-Plex A kit from Quanterix on a Simoa HD-1 Analyzer according to the manufacturer’s instructions, as previously described [36].

Laboratory technicians were blinded to all clinical and genetic information.

### Statistical analysis

Statistical analyses were performed in IBM SPSS Statistics 25 and *R*.

Demographic and clinical variables were compared between groups (symptomatic, presymptomatic, non-carrier) using Kruskal–Wallis tests for continuous variables and a Chi-square test for sex. Normality of biomarker data was assessed using Kolmogorov–Smirnov tests and visual inspection of *Q*–*Q* plots. All raw protein concentrations, both in CSF and plasma, were non-normally distributed. For CSF analytes, normal distributions were achieved after log-transformation, and we subsequently performed ANCOVAs with age and sex as covariates to compare protein concentrations between groups. For plasma complement proteins, normal distributions could not be achieved with conventional transformations (e.g. log transformation, Box–Cox transformation), and we therefore applied quantile regression, which is robust to non-normality and outliers, with age and sex as covariates. In comparisons between symptomatic mutation carriers, we also included disease duration as a covariate. Correlations between raw biomarker values and clinical and neuroimaging measures, as well as NfL and GFAP, were assessed using Spearman’s rho for presymptomatic and symptomatic mutation carriers separately. Correction for multiple comparisons was done with the Holm–Bonferroni method. We restricted correlative analyses between CSF and plasma measurements to subjects for whom the time interval between both sample collections was less than 6 months.

## Results

### Subjects

Subject characteristics of the CSF and plasma cohorts are shown in Table 1.

### CSF complement levels

We excluded 7 samples from C1q analyses (5 presymptomatic carriers, 2 non-carriers) and 12 samples from C3b analyses (5 presymptomatic and 3 symptomatic carriers, 4 non-carriers) due to duplicate CV’s > 20%. CSF C1q correlated strongly with C3b levels (*r*_s_ = 0.709, *p* < 0.001). Furthermore, C1q and C3b correlated with age at sample collection (*r*_s_ = 0.359 and *r*_s_ = 0.323; both *p* < 0.001) in the entire cohort, but not in non-carriers alone (Additional file 1: Table S2).

#### Group differences

**Fig. 1:**
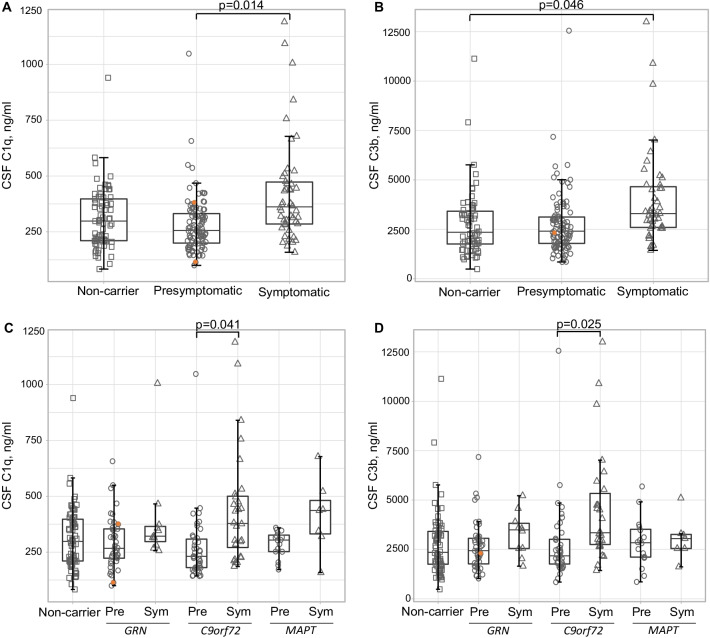
Group comparisons of CSF C1q and C3b concentration. **A** C1q in clinical groups; **B** C1q in genetic subgroups; **C** C3b in clinical groups; **D** C3b in genetic subgroups. Orange circles indicate presymptomatic carriers who developed symptoms during follow-up, one of whom was excluded from C3b analyses due to a duplicate CV > 20%. *P*-values were derived from ANCOVAs on log-transformed biomarker data with age and sex as covariates. *Pre*  presymptomatic, *Sym*  symptomatic

C1q levels were significantly higher in symptomatic mutation carriers (median 362 ng/ml (interquartile range 284–481)) than in presymptomatic carriers (256 ng/ml (199–337), *p* = 0.014), but not compared to non-carriers (298 ng/ml (210–402), *p* = 0.148) (Fig. 1a). Higher levels of C3b were found in symptomatic carriers (3295 ng/ml (2558–4734)) compared to non-carriers (2350 ng/ml (1730–3452), *p* = 0.046). However, C3b levels between symptomatic and presymptomatic carriers (2406 ng/ml (1772–3127) were not significantly different (*p* = 0.074) (Fig. 1b). After exclusion of extreme outliers (> median + 3*IQR; *n* = 1 for C1q and *n* = 5 for C3b), C3b levels were also elevated compared to presymptomatic carriers (*p* = 0.038).

Separated by genetic group, C1q and C3b levels were elevated in all symptomatic carriers, but after correction for age, group differences were only significant for *C9orf72* mutation carriers (C1q: *p* = 0.041; C3b: *p* = 0.025) (Fig. 1c, d). C1q or C3b levels did not differ between symptomatic carriers of different genetic groups (*p* = 0.351).

#### Correlative analyses

**Fig. 2:**
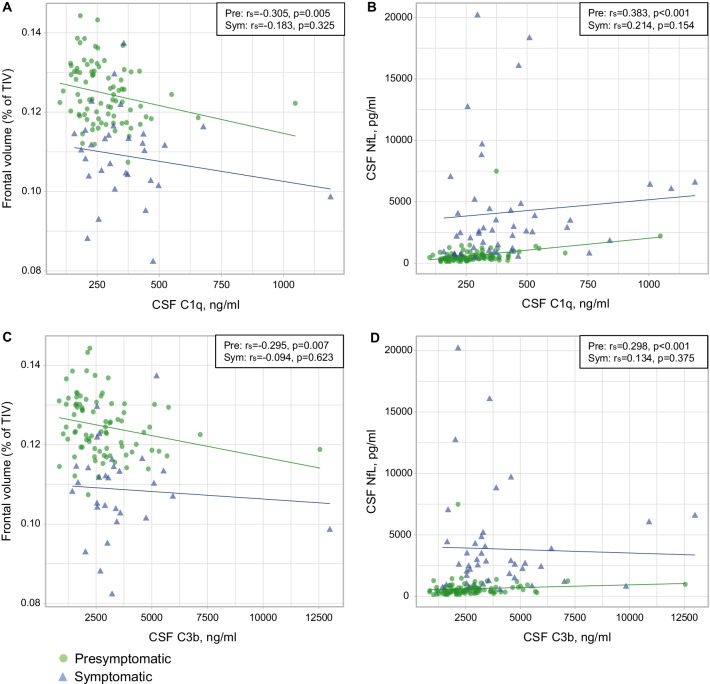
Correlations among mutation carriers between **A** CSF C1q and frontal lobe volume; **B** CSF C1q and CSF neurofilament light chain (NfL); **C** CSF C3b and frontal volume); and **D** CSF C3b and CSF NfL. *P*-values were derived from Spearman’s rho. *NfL*  neurofilament light chain, *TIV*  total intracranial volume, *Pre*  presymptomatic, *Sym*  symptomatic

In presymptomatic mutation carriers, C1q and C3b levels correlated with NfL and inversely with frontal lobe volume (Fig. 2 and Additional file 1: Table S3a). These correlations remained significant after correction for age.

In symptomatic carriers, we observed an inverse correlation between C1q and MMSE (*r*_s_ = − 0.370, *p* = 0.013) (Additional file 1: Table S4a), and C3b levels—but not C1q—were correlated with disease duration (*r*_s_ = 0.343, *p* = 0.024) (Additional file 1: Fig. S1a and 1b).

### Plasma complement levels

C3b, C5a and C9 were excluded from analyses as concentrations were below the LLOQ in 80–100% of samples; the samples with levels above the LLOQ were from all clinical groups. For some analytes, a small number of samples was excluded due to CVs > 20% (Additional file 1: Table S1).

Moderate correlations were found between most plasma analytes (Additional file 1: Table S5). Furthermore, we found positive correlations between age and almost all analytes (Additional file 1: Table S2); several of these remained significant when analyses were limited to non-carriers.

#### Group comparisons

**Fig. 3:**
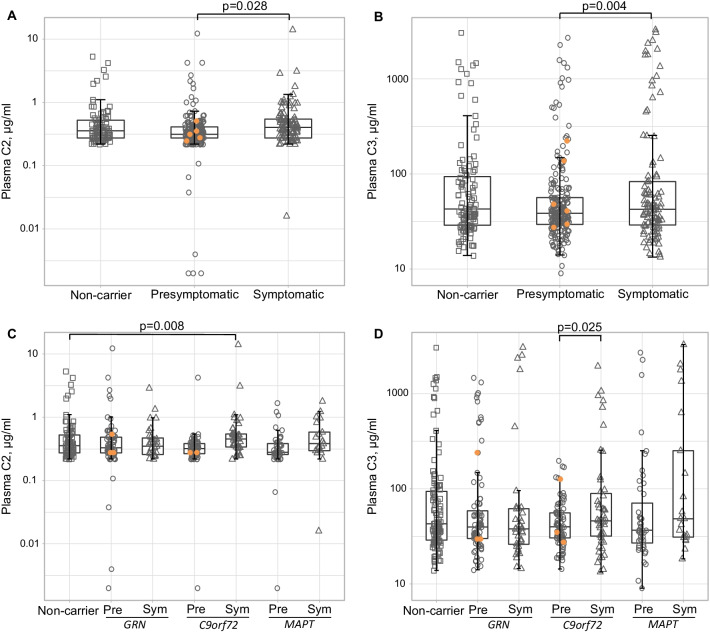
Group comparisons of plasma C2 and C3 concentration. **A** Plasma C2 in clinical groups; **B** Plasma C3 in clinical groups; **C** Plasma C2 in genetic subgroups; **D** Plasma C3 in genetic subgroups. Protein concentrations were plotted on a logarithmic scale for ease of visualisation. Orange circles indicate presymptomatic carriers who developed symptoms during follow-up, one of whom was excluded from C2 analyses due to a duplicate CV > 20%. *P*-values were derived from quantile regression models with age and sex as covariates. *Pre*  presymptomatic, *Sym*  symptomatic

Symptomatic mutation carriers had significantly higher levels of plasma C2 and C3 than presymptomatic carriers (Table 2, Fig. 3a, b).

**Table 2:** Plasma complement levels per clinical group

	Non-carriers	Presymptomatic carriers	Symptomatic carriers	*p*
C2	0.358 (0.274–0.532)	0.332 (0.274–0.431)	0.411 (0.319–0.548)	0.006*
C4b	9.97 (8.00–12.3)	9.92 (8.15–12.8)	10.8 (8.88–14.3)	0.913
C5	29.1 (21.6–35.8)	27.0 (21.7–35.2)	31.0 (25.1–38.3)	0.358
Factor D	3.57 (2.99–4.37)	3.46 (2.69–4.15)	4.19 (3.50–5.28)	0.481
MBL	2.44 (0.841–4.20)	2.01 (0.860–4.12)	1.71 (0.763–4.41)	0.710
Factor I	39.6 (32.9–46.6)	37.9 (32.8–45.2)	40.9 (35.0–47.0)	0.863
C1q	71.4 (64.5–81.2)	71.2 (60.6–80.1)	68.7 (62.2–76.2)	0.636
C3	44.8 (29.9–98.2)	39.9 (29.9–58.8)	45.5 (30.5–102)	0.047**
C4	293 (254–360)	297 (252–348)	294 (254–349)	0.577
Factor B	168 (147–211)	167 (146–205)	170 (143–202)	0.772
Factor H	250 (213–295)	250 (212–288)	254 (228–285)	0.849

All concentrations are expressed as medians (interquartile range) in µg/ml. *P*-values are derived from quantile regression models with age and sex as covariates. *MBL* mannose-binding lectin. *Pairwise comparisons: symptomatic vs presymptomatic carriers: *p* = 0.028. **Pairwise comparisons: symptomatic vs presymptomatic carriers: *p* = 0.004

Separated by genetic subgroup, elevated levels of C2 and C3 were observed in symptomatic *C9orf72* and *MAPT*—but not *GRN*—mutation carriers, reaching statistical significance in *C9orf72* (Fig. 3c, d). No significant differences were observed in C2 or C3 levels between symptomatic carriers of different genetic subgroups (C2: *p* = 0.425; C3: *p* = 0.512).

#### Correlative analyses

**Fig. 4:**
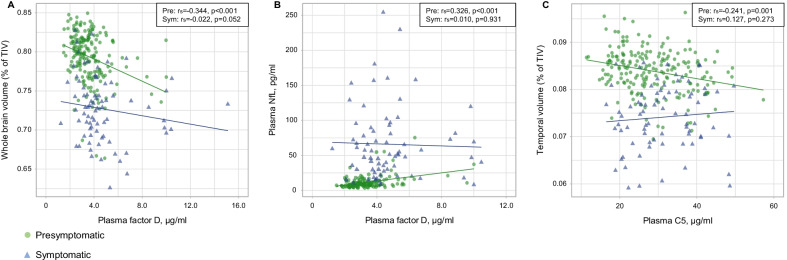
Correlations among mutation carriers between **A** plasma factor D and whole brain volume; **B** plasma factor D and plasma neurofilament light chain (NfL); and **C** plasma C5 and temporal lobe volume. *P*-values were derived from Spearman’s rho. *NfL*  neurofilament light chain, *TIV*  total intracranial volume, *Pre*  presymptomatic, *Sym*  symptomatic

In the presymptomatic stage, inverse correlations were observed between several complement proteins and regional grey matter volume. The strongest correlations were observed for factor D with WBV (*r*_s_ = − 0.344, *p* < 0.001), temporal volume (*r*_s_ = − 0.271, *p* < 0.001) and volume of the cingulate gyrus (*r*_s_ = − 0.262, *p* < 0.001), and for C5 with temporal volume (*r*_s_ = − 0.241, *p* = 0.001), which remained significant after correction for age. Furthermore, C4b, C5 and factor D were positively correlated with NfL and GFAP (Fig. 4, Additional file 1: Tables S3b, 6).

In the symptomatic stage, C2, C3 and factor D were inversely correlated with WBV and volume of the temporal and parietal lobes, cingulate gyrus and insula, and C2, C3, factor D, factor I and factor H were correlated with CDR® + NACC FTLD-SB score (Additional file 1: Tables S3b, 4b). Furthermore, we found positive correlations with disease duration for C2 (*r*_s_ = 0.279, *p* = 0.006), factor D (*r*_s_  = 0.239, *p* = 0.015) and factor I (*r*_s_  = 0.202, *p* = 0.039).

### Correlation between CSF and plasma C1q levels

CSF and plasma C1q levels were not correlated among 147 subjects with matched CSF and plasma samples (*r*_s_  = 0.092, *p* = 0.266; mean time interval between CSF and plasma: 13 days). Restricting analyses to samples collected on the same day similarly revealed no correlation (*r*_s_  = 0.065, *p* = 0.543, *n* = 91).

### CSF and plasma complement levels in converters

In the seven presymptomatic carriers who were diagnosed with FTD during follow-up (‘converters’), no relationship between CSF or plasma complement levels and time to symptom onset was observed (Additional file 1: Table S7).

## Discussion

This large, international study demonstrated elevated levels of several complement proteins in CSF and plasma in the symptomatic stages of genetic FTD, as well as correlations with various measures of disease severity. Our findings provide in vivo evidence of an inflammatory component in FTD and could aid therapeutic trials aimed at modulation of the immune response.

The elevated levels of C1q and C3b in CSF of symptomatic mutation carriers probably reflect increased local synthesis of complement proteins by glial cells and neurons, as has previously been reported in neurodegeneration [18, 44–47]. C1q is the initiator molecule of the classical pathway, and its binding to immune complexes, apoptotic cells and various other stimuli triggers a cascade of protein reactions to generate C3b [14]. C3b is one of the primary complement opsonins, and its accumulation on synapses and subsequent recognition by phagocytic microglia is thought to underlie the synapse loss observed early in the neurodegenerative process [18–20]. If direct associations can be confirmed, CSF C1q and C3b might provide a means to monitor complement-mediated synaptic pruning and measure the effect of complement-directed therapeutics [44]. C3b also elicits generation of the cytotoxic terminal C5b-C9 complex (TCC) [14]. It would be interesting to expand on our results by measuring CSF complement proteins directly implicated in the TCC, as well as regulatory factors, a decrease of which might further amplify aberrant complement activation [48].

In symptomatic mutation carriers, plasma measurements revealed elevated levels of C2, a component of the classical pathway, and C3. Rather than reflecting overflow from the central nervous system (CNS), these findings might reflect a systemic immune response, which could in turn contribute to neuroinflammation by passing through the (compromised) blood–brain barrier [44, 49]. The lack of a correlation between CSF and plasma C1q suggests that systemic and local complement activation might not occur simultaneously and indicates that plasma complement measurement is not a suitable surrogate for CSF. Investigation of CSF–plasma associations of other complement proteins besides C1q might confirms this, in which case brain-derived extracellular vesicles could provide a better peripheral measure of CNS complement activation [25, 28]. Furthermore, since consumption of intact complement components (e.g. C3, C4 and C5) can paradoxically reduce plasma levels during strong complement activation [38], future measurements of activated fragments (e.g. C3a, C3b and components of the TCC) in FTD might provide more robust measures of peripheral complement activation.

In genetic subgroup analyses, the elevated complement protein levels in CSF and plasma remained statistically significant only in *C9orf72* mutation carriers. Interestingly, *C9orf72*^−/−^ mice have been shown to have upregulated interferon-β expression, increased microglial activation, and excessive synaptic pruning compared to wild-type mice [50]. In vivo administration of interferon-β drives microglial activation and complement C3-dependent synapse elimination [51]. *C9orf72* deficiency might thus promote microglial activation through interferon-β, in turn leading to synaptic elimination by complement activation. Alternatively, since complement activation has also been reported in cell and animal models of *GRN* and *MAPT* mutations [17, 21, 22, 52], the lack of significant differences in *GRN*- and *MAPT*-related FTD might instead reflect a lack of statistical power given the smaller sample size of these genetic subgroups. The elevated levels of complement proteins in various other neurodegenerative diseases similarly point towards a general rather than gene- or disease-specific upregulation of the complement system [21–30]. Future studies comparing complement levels in genetic and sporadic forms of FTD and associated clinical subtypes might further elucidate potential gene-specific effects.

In presymptomatic mutation carriers, CSF and plasma complement levels correlated with regional grey matter volume and NfL. These correlations remained significant even after correction for age, and suggest that complement activation might occur in the late-presymptomatic stage in conjunction with early brain atrophy. Accordingly, elevated complement levels have been observed in presymptomatic genetic AD [27, 53] and mild cognitive impairment [26, 28]. In AD mouse models, complement aggregation is observed prior to plaque formation [17, 18, 52]. Despite applying statistical correction for age, the lack of group differences in complement levels between presymptomatic carriers and non-carriers could partly be due to including carriers of all ages, and thus time to symptom onset was highly variable.

CSF and plasma complement levels showed substantial overlap between groups, which has also been reported in AD [26, 27, 54] and precludes their use as diagnostic biomarkers. NfL may be a more powerful tool to distinguish symptomatic from presymptomatic mutation carriers [34]. The large variability in complement levels, which was observed even among non-carriers, suggests that within-individual changes in complement levels may be more informative for disease monitoring than single measurements. The lack of correlation in symptomatic mutation carriers between CSF complement factors and most disease severity measures, including brain atrophy, NfL, GFAP and MMSE, indicates that complement levels probably do not increase linearly as the disease progresses. Instead, in line with the dynamic nature of neuroinflammation [3], they might fluctuate depending on the disease stage [55]. Longitudinal studies of CSF and plasma complement factors, including a larger number of converters, might elucidate their dynamics over the course of FTD.

Strengths of this study include the very large, well-characterised genetic FTD cohort with corresponding clinical and neuroimaging data. In plasma, we measured a broad range of complement proteins covering all three activation pathways as well as various regulatory molecules. The strong correlations between CSF C1q and C3b, as well as between the various plasma complement factors, support the validity of our results.

The findings presented in this study must be viewed in light of some limitations. Our plasma complement measurements could have been affected by various confounding factors, including body mass index, hypertension, diabetes mellitus and (asymptomatic or low-grade) inflammatory processes [38, 56]. Although we excluded subjects with known auto-immune diseases, we cannot rule out the presence of other inflammatory conditions, such as infections. Future research should include a blood panel to check for infectious parameters at the time of sample collection. Furthermore, complement proteins are sensitive to variability in pre-analytical parameters [57], which could have affected our results, despite following standardised protocols for sample collection and processing. Finally, we were unable to quantify plasma C3b, C5a and C9 levels, presumably due to very low concentrations, highlighting the need for more sensitive assays.

## Conclusions

In conclusion, we provide in vivo evidence of complement activation in genetic FTD, which might already occur in late-presymptomatic stages in conjunction with neuronal loss. Future longitudinal studies could elucidate at which stage of disease complement levels start to change, and might reveal their potential value as monitoring biomarkers [44].

## Supplementary Information

**Additional file 1: Table S1.** Number of samples for each of the analytes in CSF and plasma. **Table S2.** Correlations between complement proteins and age. **Table S3.** Correlations between grey matter volume and (a) CSF and (b) plasma complement protein concentration. **Table S4.** Correlations between clinical measures of disease severity and (a) CSF and (b) plasma complement proteins. **Table S5.** Correlations between plasma complement factors. **Table S6.** Correlations between plasma complement proteins, neurofilament light chain (NfL) and glial fibrillary acidic protein (GFAP). **Table S7.** Complement protein levels of seven presymptomatic carriers who became symptomatic during follow-up (‘converters’). **Figure S1.** Correlations between CSF C1q, C3b and disease duration. P-values were derived from Spearman’s rho.

## Data Availability

The raw data of this project are part of GENFI. De-identified patient data can be accessed upon reasonable request to genfi@ucl.ac.uk.

## Abbreviations

AD: Alzheimer’s disease
ALS: Amyotrophic lateral sclerosis
bvFTD: Behavioural variant FTD
*C9orf72*: Chromosome 9 open reading frame 72
CDR® + NACC FLTD-SB: Clinical Dementia Rating scale plus NACC frontotemporal lobar degeneration—sum of boxes
CNS: Central nervous system
CSF: Cerebrospinal fluid
CV: Coefficient of variation
FTD: Frontotemporal dementia
GENFI: Genetic frontotemporal dementia initiative
GFAP: Glial fibrillary acidic protein
*GRN*: Granulin
LLOQ: Lower limit of quantification
*MAPT*: Microtubule-associated protein tau
MBL: Mannose-binding lectin
MMSE: Mini Mental State Examination
NfL: Neurofilament light chain
PPA: Primary progressive aphasia
PSP: Progressive supranuclear palsy
TCC: Terminal complement complex
TIV: Total intracranial volume
ULOQ: Upper limit of quantification
WBV: Whole brain volume
