# Supplementary material for: Elevated CSF and plasma complement proteins in genetic frontotemporal dementia: results from the GENFI study
Source: J Neuroinflammation. 2022 Sep 5;19:217. doi: 10.1186/s12974-022-02573-0 (PMC9446850; doi:10.1186/s12974-022-02573-0)
Supplement: Supplementary file 1 — Additional file 1: Table S1. Number of samples for each of the analytes in CSF and plasma. Table S2. Correlations between complement proteins and age. Table S3. Correlations between grey matter volume and (a) CSF and (b) plasma complement protein concentration. Table S4. Correlations between clinical measures of disease severity and (a) CSF and (b) plasma complement proteins. Table S5. Correlations between plasma complement factors. Table S6. Correlations between plasma complement proteins, neurofilament light chain (NfL) and glial fibrillary acidic protein (GFAP). Table S7. Complement protein levels of seven presymptomatic carriers who became symptomatic during follow-up (‘converters’). Figure S1. Correlations between CSF C1q, C3b and disease duration. P-values were derived from Spearman’s rho. [file 12974_2022_2573_MOESM1_ESM.pdf]

Additional file 1: Supplementary tables

Table S1. Number of samples for each of the analytes in CSF and plasma.

Analyte	Samples measured		Samples included in statistical analyses		
	N	N (CV>20%)	N	N <LLOQ or >ULOQ	Mean %CV (range)
CSF					
C1q	224	7	217	0	5.3 (0.1-20)
C3b	224	12	212	0	6.6 (0-19)
NfL	224	0	224	0	3.2 (0-16)
Plasma					
C2	427	48	379	17	6.9 (0-20)
C4b	427	2	425	0	2.6 (0-18)
C5	427	2	425	0	2.8 (0-13)
Factor D	427	4	423	3	2.8 (0-20)
MBL	427	2	425	1	2.1 (0-20)
Factor I	427	1	426	0	2.4 (0-20)
C1q	431	1	430	3	2.2 (0-11)
C3	431	1	430	1	2.6 (0-18)
C4	431	0	431	0	1.7 (0-15)
Factor B	431	2	429	3	2.1 (0-17)
Factor H	431	0	431	0	1.9 (0-13)
NfL	276	1	275	0	4.5 (0-18)
GFAP	276	0	276	0	3.3 (0-20)

Samples with a duplicate coefficient of variation (CV) >20% were re-measured or, if materials were insufficient, excluded from statistical analyses. For sample concentrations outside of the range of quantification, we imputed the lower or upper limits of quantification (LLOQ and ULOQ). NfL, neurofilament light chain; MBL, mannose-binding lectin; GFAP, glial fibrillary acidic protein.

Table S2. Correlations between complement proteins and age.

		Entire cohort	Non-carriers
CSF			
C1q	r_s	0.359	0.188
	p	<0.001	0.115
C3b	r_s	0.323	0.169
	p	<0.001	0.161
Plasma			
C2	r_s	0.150	0.077
	p	0.003	0.464
C4b	r_s	0.177	0.183
	p	<0.001	0.058
C5	r_s	0.255	0.327
	p	<0.001	0.001
Factor D	r_s	0.411	0.255
	p	<0.001	0.007
MBL	r_s	-0.057	0.000
	p	0.244	0.999
Factor I	r_s	0.215	0.389
	p	<0.001	<0.001
C1q	r_s	0.058	0.100
	p	0.234	0.292
C3	r_s	-0.064	-0.223
	p	0.188	0.018
C4	r_s	0.096	0.192
	p	0.047	0.042
Factor B	r_s	0.094	0.176
	p	0.052	0.064
Factor H	r_s	0.163	0.184
	p	0.001	0.052

MBL, mannose-binding lectin.

Table S3. Correlations between grey matter volume and (a) CSF and (b) plasma complement protein concentration.

Supplementary table 3a: CSF complement and grey matter volume								
		WBV	Frontal lobe	Temporal lobe	Parietal lobe	Occipital lobe	Cingulate gyrus	Insula
Presymptomatic carriers								
C1q	r_s	-0.141	-0.305	-0.109	-0.179	-0.121	-0.158	-0.162
	p	0.203	0.005*	0.328	0.106	0.275	0.153	0.143
C3b	r_s	-0.143	-0.295	-0.043	-0.123	-0.067	-0.225	-0.182
	p	0.198	0.007*	0.702	0.268	0.547	0.041	0.099
Symptomatic carriers								
C1q	r_s	-0.226	-0.183	-0.177	-0.337	-0.426	-0.095	-0.367
	p	0.221	0.325	0.340	0.064	-0.017	0.611	0.042
C3b	r_s	-0.211	-0.094	-0.171	-0.251	-0.197	0.002	-0.136
	p	0.263	0.623	0.367	0.181	0.296	0.990	0.472

Supplementary table 3b: Plasma complement and grey matter volume								
		WBV	Frontal lobe	Temporal lobe	Parietal lobe	Occipital lobe	Cingulate gyrus	Insula
Presymptomatic carriers								
C2	r_s	0.051	0.010	0.002	0.122	0.040	0.053	-0.010
	p	0.496	0.892	0.980	0.106	0.592	0.484	0.896
C4b	r_s	-0.073	-0.046	-0.166	-0.044	-0.080	-0.005	-0.131
	p	0.308	0.519	0.019	0.536	0.263	0.945	0.066
C5	r_s	-0.174	-0.101	-0.241	-0.161	-0.173	-0.040	-0.113
	p	0.014	0.160	0.001*	0.023	0.015	0.581	0.118
F. D	r_s	-0.344	-0.226	-0.271	-0.230	-0.189	-0.262	-0.144
	p	<0.001*	0.001*	<0.001*	0.001*	0.008	<0.001*	0.044
MBL	r_s	0.122	0.123	0.156	0.146	0.173	0.104	0.145
	p	0.088	0.086	0.029	0.041	0.015	0.147	0.041
F. I	r_s	-0.152	-0.129	-0.208	-0.126	-0.162	-0.062	-0.094
	p	0.032	0.070	0.003*	0.078	0.023	0.386	0.0187
C1q	r_s	-0.069	-0.073	-0.060	-0.013	-0.178	0.012	0.011
	p	0.334	0.303	0.400	0.069	0.012	0.866	0.872
C3	r_s	0.147	-0.038	0.039	0.030	0.062	0.089	0.019
	p	0.038	0.592	0.582	0.671	0.384	0.212	0.789
C4	r_s	-0.052	-0.095	-0.174	-0.060	-0.077	-0.059	-0.091
	p	0.465	0.183	0.014	0.402	0.282	0.407	0.198
F. B	r_s	-0.090	-0.123	-0.175	-0.086	-0.153	-0.024	-0.065
	p	0.205	0.082	0.013	0.229	0.031	0.733	0.362
F. H	r_s	-0.156	-0.176	-0.180	-0.201	-0.229	-0.054	-0.141
	p	0.027	0.012*	0.011*	0.004*	0.001*	0.447	0.046
Symptomatic carriers								
C2	r_s	-0.237	-0.258	-0.231	-0.395	-0.262	-0.357	-0.223
	p	0.048	0.031	0.054	0.001*	0.028	0.002*	0.064
C4b	r_s	0.114	0.75	0.038	0.045	0.038	-0.047	0.101
	p	0.324	0.516	0.745	0.698	0.742	0.684	0.380
C5	r_s	0.272	0.193	0.127	0.058	0.006	0.094	0.195
	p	0.017	0.092	0.273	0.619	0.958	0.415	0.090
F. D	r_s	-0.222	-0.190	-0.352	-0.385	-0.201	-0.353	-0.209
	p	0.052	0.098	0.002*	0.001*	0.080	0.002*	0.068
MBL	r_s	-0.051	-0.067	0.040	-0.125	-0.152	-0.105	-0.068

	p	0.657	0.564	0.731	0.277	0.187	0.363	0.557
F. I	r_s	-0.084	-0.101	-0.253	-0.035	-0.086	-0.160	-0.116
	p	0.469	0.381	0.026	0.764	0.459	0.163	0.315
C1q	r_s	0.070	0.093	-0.062	0.079	0.060	0.063	-0.079
	p	0.564	0.423	0.595	0.498	0.607	0.587	0.500
C3	r_s	-0.409	-0.386	-0.261	-0.270	-0.146	-0.443	-0.363
	p	<0.001	0.001*	0.022*	0.018	0.205	<0.001*	0.001*
C4	r_s	0.133	0.049	0.036	0.107	0.144	-0.018	0.050
	p	0.250	0.675	0.756	0.352	0.210	0.876	0.664
F. B	r_s	0.148	0.060	0.031	0.167	0.170	0.098	0.003
	p	0.198	0.607	0.786	0.147	0.138	0.398	0.982
F. H	r_s	-0.014	-0.128	-0.077	0.089	0.081	-0.083	-0.097
	p	0.901	0.266	0.506	0.440	0.481	0.475	0.401

All grey matter volumes were corrected for total intracranial volume. * indicates a result that remained significant after Holm-Bonferroni correction for each of the analytes separately. WBV, whole brain volume; F. D, Factor D; MBL, mannose-binding lectin; F. I, Factor I; F. B, Factor B; F. H, factor H.

Table S4. Correlations between clinical measures of disease severity and (a) CSF and (b) plasma complement proteins.

Supplementary table 4a. CSF complement and clinical disease severity scores			
		MMSE	CDR® + NACC FTLD-SB
Presymptomatic carriers			
C1q	r_S	-0.370	0.229
	p	0.013	0.233
C3b	r_S	-0.298	0.354
	p	0.059	0.070
Symptomatic carriers			
C1q	r_S	0.007	0.017
	p	0.946	0.877
C3b	r_S	0.027	0.065
	p	0.794	0.543

Supplementary table 4b. Plasma complement and clinical disease severity scores			
		MMSE	CDR plus NACC FTLD-SB
Presymptomatic carriers			
C2	r_S	0.092	-0.086
	p	0.212	0.291
C4b	r_S	-0.092	0.081
	p	0.189	0.291
C5	r_S	-0.102	0.017
	p	0.144	0.823
Factor D	r_S	-0.105	0.073
	p	0.132	0.348
MBL	r_S	-0.025	0.111
	p	0.720	0.149
Factor I	r_S	-0.033	0.049
	p	0.638	0.523
C1q	r_S	-0.065	-0.073
	p	0.349	0.342
C3	r_S	0.074	-0.141
	p	0.284	0.064
C4	r_S	-0.021	0.001
	p	0.762	0.987
Factor B	r_S	0.032	-0.005
	p	0.600	0.947
Factor H	r_S	-0.069	-0.010
	p	0.322	0.893
Symptomatic carriers			
C2	r_S	-0.056	0.369
	p	0.615	0.003
C4b	r_S	0.112	0.045
	p	0.291	0.707
C5	r_S	-0.076	-0.028
	p	0.473	0.818
Factor D	r_S	-0.204	0.270
	p	0.054	0.022
MBL	r_S	-0.237	0.077
	p	0.023	0.523
Factor I	r_S	-0.094	0.193

	p	0.374	0.104
C1q	r_s	-0.188	0.175
	p	0.075	0.141
C3	r_s	-0.118	0.446
	p	0.266	<0.001
C4	r_s	-0.166	0.217
	p	0.117	0.067
		MMSE	CDR plus NACC FTLD-SB
Factor B	r_s	-0.200	0.270
	p	0.057	0.022
Factor H	r_s	-0.187	0.340
	p	0.076	0.003

* indicates a result that remained significant after Holm-Bonferroni correction. MMSE, Mini Mental State Examination; CDR® + NACC FTLD-SB, Clinical Dementia Rating scale plus NACC Frontotemporal Lobar Degeneration – sum of boxes; F. D, Factor D; MBL, mannose-binding lectin; F. I, Factor I; F. B, Factor B; F. H, factor H.

Table S5. Correlations between plasma complement factors.

		C2	C4b	C5	F.D	MBL	F.I	C1q	C3	C4	F.B
C2	r_s	-									
	p	-									
C4b	r_s	0.276	-								
	p	0.008	-								
C5	r_s	0.077	0.593	-							
	p	0.468	<0.001	-							
F.D	r_s	0.241	0.244	0.208	-						
	p	0.021	0.011	0.030	-						
MBL	r_s	0.247	-0.040	0.047	0.270	-					
	p	0.018	0.678	0.626	0.005	-					
F.I	r_s	0.326	0.349	0.560	0.440	0.126	-				
	p	0.002	<0.001	<0.001	<0.001	0.193	-				
C1q	r_s	-0.080	0.052	0.298	0.134	0.069	0.386	-			
	p	0.450	0.595	0.002	0.164	0.478	<0.001	-			
C3	r_s	0.383	0.044	-0.122	-0.041	0.048	0.078	0.224	-		
	p	<0.001	0.655	0.208	0.673	0.617	0.418	0.018	-		
C4	r_s	0.090	0.269	0.450	0.222	0.019	0.622	0.495	0.213	-	
	p	0.396	0.005	<0.001	0.021	0.849	<0.001	<0.001	0.025	-	
F.B	r_s	0.103	0.125	0.456	0.156	0.115	0.629	0.547	0.176	0.831	-
	p	0.333	0.199	<0.001	0.108	0.237	<0.001	<0.001	0.069	<0.001	-
F.H	r_s	-0.004	0.181	0.544	0.170	0.005	0.539	0.618	0.182	0.777	0.744
	p	0.971	0.061	<0.001	0.078	0.960	<0.001	<0.001	0.056	<0.001	<0.001

F. D, Factor D; MBL, mannose-binding lectin; F. I, Factor I; F. B, Factor B; F. H, factor H.

Table S6. Correlations between plasma complement proteins, neurofilament light chain (NfL) and glial fibrillary acidic protein (GFAP).

		NfL	GFAP
Presymptomatic carriers			
C2	r_s	-0.107	-0.054
	p	0.254	0.560
C4b	r_s	0.223	0.221
	p	0.010*	0.010*
C5	r_s	0.219	0.196
	p	0.011	0.023*
Factor D	r_s	0.326	0.197
	p	<0.001*	0.022*
MBL	r_s	-0.106	-0.085
	p	0.228	0.331
Factor I	r_s	-0.031	0.046
	p	0.719	0.597
C1q	r_s	-0.044	0.051
	p	0.615	0.561
C3	r_s	-0.191	-0.125
	p	0.027	0.148
C4	r_s	0.049	0.100
	p	0.571	0.247
Factor B	r_s	0.030	0.123
	p	0.731	0.158
Factor H	r_s	0.041	0.103
	p	0.638	0.234
Symptomatic carriers			
C2	r_s	-0.236	-0.123
	p	0.057	0.325
C4b	r_s	-0.145	-0.030
	p	0.223	0.805
C5	r_s	0.029	0.146
	p	0.811	0.221
Factor D	r_s	0.010	0.179
	p	0.931	0.135
MBL	r_s	-0.111	0.056
	p	0.354	0.640
Factor I	r_s	-0.174	-0.002
	p	0.145	0.987
C1q	r_s	-0.089	-0.002
	p	0.459	0.989
C3	r_s	-0.154	-0.074
	p	0.198	0.535
C4	r_s	-0.023	-0.081
	p	0.848	0.497
Factor B	r_s	0.046	-0.008
	p	0.703	0.948
Factor H	r_s	0.125	0.021
	p	0.295	0.859

* indicates a result that remained significant after Holm-Bonferroni correction. MBL, mannose-binding lectin.

Table S7. Complement protein levels of seven presymptomatic carriers who became symptomatic during follow-up ('converters').

	Genetic subgroup	Time to symptom onset, years	CSF C1q, ng/ml	CSF C3b, ng/ml	Plasma C2, pg/ml	Plasma C3, pg/ml
1	*GRN*	2.2	113	878	0.22	32.4
2	*GRN*	-0.4*	-	-	0.31	29.6
3	*C9orf72*	1	-	-	0.33	196
4	*GRN*	0.4	-	-	0.61	321
5	*C9orf72*	1.7	-	-	-	35.6
6	*C9orf72*	0.5	-	-	0.27	22.0
7	*GRN*	0.2	374	2147	-	-

Time to symptom onset was based on retrospective estimations by the primary caregiver.*Symptoms were retrospectively estimated to have begun before the study visit, but at the time, the subject was considered presymptomatic.
